# Elevated glycine detected on in vivo magnetic resonance spectroscopy in a breast cancer patient: case report and literature review

**DOI:** 10.1259/bjrcr.20190090

**Published:** 2020-02-12

**Authors:** Almir G.V. Bitencourt, Katja Pinker, Sunitha Thakur

**Affiliations:** 1 Department of Radiology, Breast Imaging Service, Memorial Sloan Kettering Cancer Center, New York, NY, USA; 2 Department of Imaging, A.C.Camargo Cancer Center, São Paulo, SP, Brazil; 3 Department of Medical Physics, Memorial Sloan Kettering Cancer Center, New York, NY, USA

## Abstract

Magnetic resonance spectroscopy (MRS) is a promising non-invasive diagnostic method that can detect and quantify endogenous tissue metabolites. High glycine levels obtained from *ex vivo* breast MRS have been associated with poor prognosis; however, glycine evaluation has not been reported regarding *in vivo* MRS. We report our finding in a breast cancer patient in whom pre-treatment but not post-treatment *in vivo* MRS showed elevated glycine and discuss the implications of this finding.

## Clinical presentation

A 54-year-old perimenopausal female complained of a mass in the left breast. Physical examination showed a 3-cm mass in the left upper inner quadrant; there were no suspicious skin or nipple changes and no palpable lymphadenopathy. Bilateral mammogram showed a 2-cm spiculated mass in the same location which was nine centimeters away from the nipple, a 1-cm satellite nodule just deep to the main tumor, and pleomorphic calcifications spanning 5 cm. Stereotactic-guided biopsy yielded poorly differentiated invasive ductal carcinoma as well as high-grade, solid-type ductal carcinoma *in situ*. At immunohistochemistry, the invasive carcinoma was determined to be *ER*/*PR* negative and *HER2* positive (3 + result on the Hercept test). Sentinel lymph node biopsy yielded micrometastasis in two axillary lymph nodes.

## Investigations/Imaging findings

**Figure 1.**
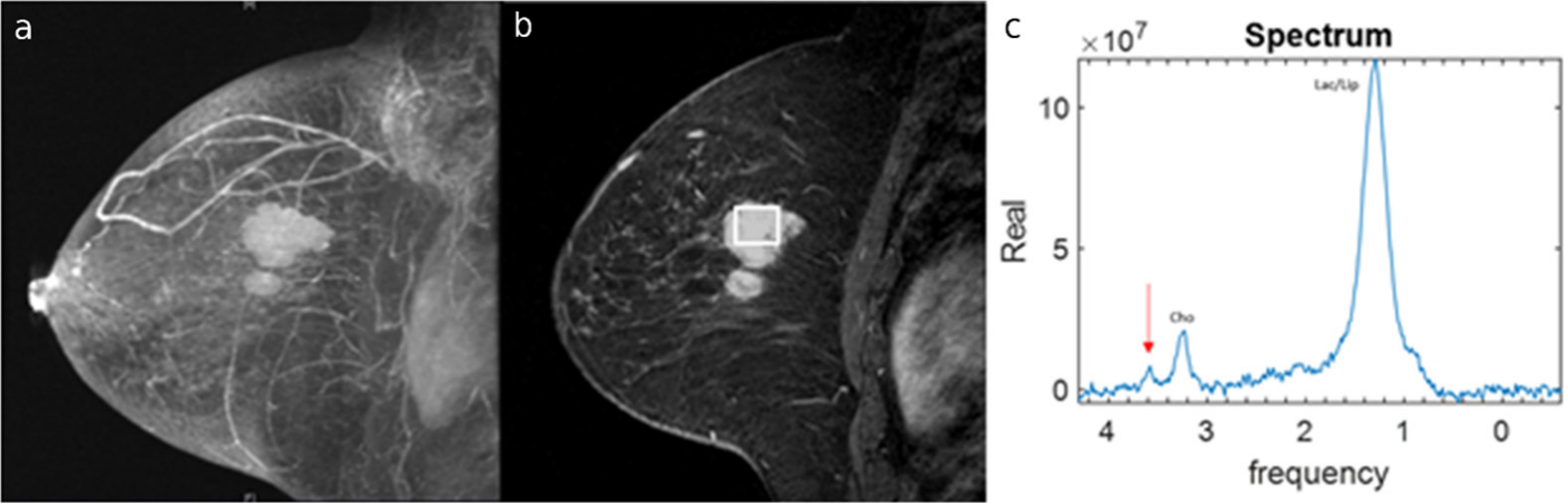
Pretreatment breast MRI in a biopsy-proven invasive ductal carcinoma in left breast of a 54-year-old female. (a) 3D maximum intensity projection reconstructed sagittal post-contrast fat-suppressed *T*_1_ weighted MR image of left breast demonstrating two adjacent masses in the upper quadrants. (b) Region-of-interest (ROI) placement for MR spectroscopy analysis. (c) MRS demonstrates a glycine peak at a frequency of 3.56 ppm (arrow) adjacent to the choline (Cho) peak.

Pretreatment bilateral breast MRI, performed on a 1.5 T device (LX or Excite; GE Medical Systems, Milwaukee, WI), demonstrated two adjacent irregular masses spanning 4 cm in the posterior third of the breast in the left upper inner quadrant, (Figure 1a). A region of interest was positioned in the larger mass (Figure 1b). Magnetic resonance spectroscopy (MRS) spectrum representative of the region of interest demonstrated a choline (Cho) peak, which is expected in malignant tumors, as well as a glycine peak (Figure 1c).

## Treatment

**Figure 2.**
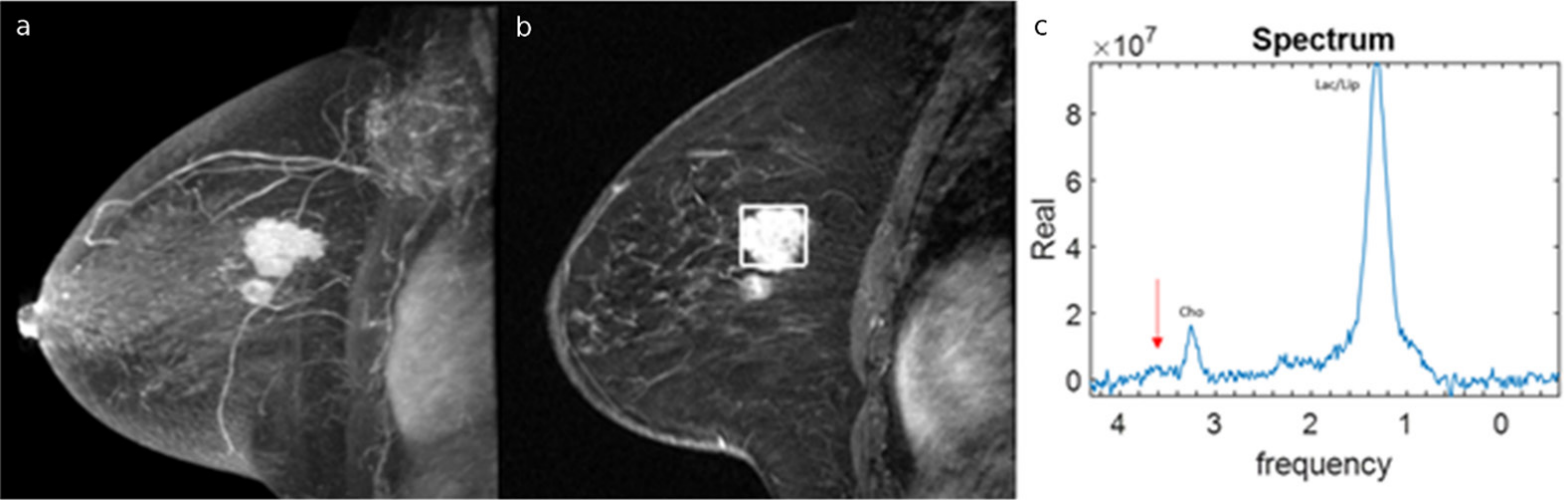
Post-treatment breast MRI performed 24 h after the first neoadjuvant chemotherapy cycle. (a) 3D maximum intensity projection reconstructed sagittal post-contrast fat-suppressed *T*_1_ weighted MR image of left breast demonstrating no morphological changes in the two adjacent masses in the upper quadrants. (b) Region-of-interest (ROI) placement for MR spectroscopy analysis. (c) MR spectrum demonstrates the absence of the glycine peak previously seen on pre-treatment breast MRI (arrow), while no significative changes can be observed in the choline peak.

The patient received neoadjuvant chemotherapy (NAC) consisting of four cycles of dose-dense AC (adriamycin and cyclophosphamide) followed by 12 cycles of weekly Taxol, along with Herceptin and Lapatinib therapy. Post-treatment breast MRI performed 24 h after the first NAC cycle demonstrated the same morphologic findings as the pre-treatment MRI; the post-treatment MRS spectrum demonstrated the absence of the glycine peak previously observed (Figure 2).

## Outcome / Follow-Up

**Figure 3.**
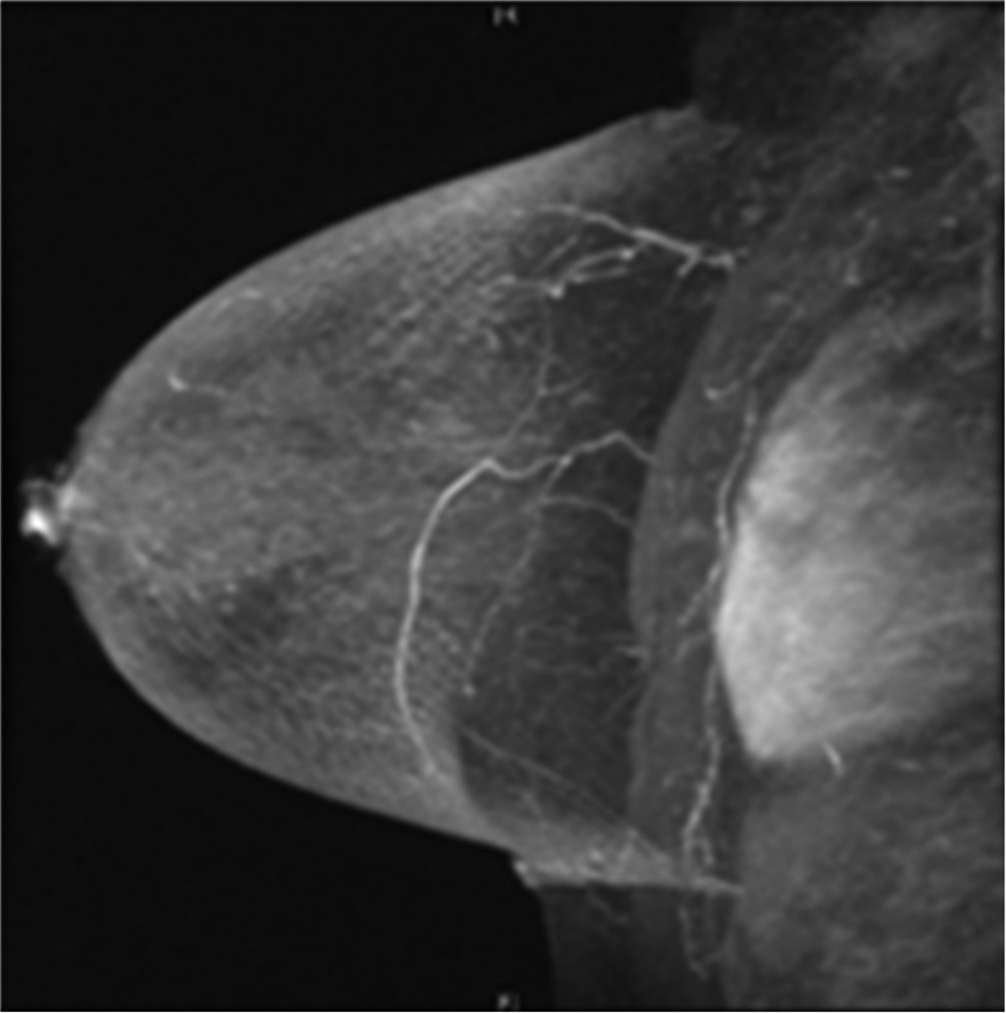
3D maximum intensity projection reconstructed sagittal post-contrast fat-suppressed *T*_1_ weighted MRI of the left breast performed after the completion of neoadjuvant chemotherapy demonstrating no residual lesion.

At the completion of NAC, breast MRI demonstrated no residual lesion (Figure 3). The patient underwent left breast local excision with MRI-guided needle localization. Pathology showed treatment effect without residual carcinoma, consistent with complete pathologic response.

## Discussion

MRS is a promising non-invasive diagnostic method that can detect and quantify endogenous tissue metabolites.^1^ In breast cancer patients, the total choline signal measured *in vivo* has been used to differentiate malignant from benign lesions, determinate tumor aggressiveness, and assess response to treatment.^2^

Recently, other metabolites besides choline have also been investigated to improve the diagnostic accuracy of breast MRS While glycine concentrations have not been described in breast carcinomas using *in vivo* MRS, authors of several studies using *ex vivo* HRMAS MRS have suggested that high glycine concentrations in breast cancer can serve as a marker of poor prognosis.^3–8^ For instance, Sitter et al and Choi et al have reported finding higher concentrations of glycine in patients with a poor prognosis based on tumor size, spread to axillary lymph nodes, and expression of hormone receptors.^6,8^ In a study comparing the metabolic profiles on high-resolution magic angle spinning (HRMAS) MRS with other conventional prognostic imaging biomarkers, Yoon et al found that higher levels of glycine correlated with the patient group with low apparent diffusion coefficient (ADC) on diffusion-weighted MR imaging as well as the patient group with high standard uptake value (SUV) on 18F-fluorodeoxyglucose positron emission tomography-CT (18F-FDG PET-CT).^4^ Of note, higher levels of glycine may be more common in *HER2* overexpressed tumors,^3,5^ which is supported by the present case study in which the patient had a *HER2* positive tumor.

While *in vivo* MRS has been used for the early assessment of response to NAC based on the changes in tumor choline levels,^9^ glycine may also be promising for early assessment of treatment response. Cao et al (2012) assessed whether breast cancer metabolic profiles using *ex vivo* HRMAS MRS can predict treatment response to NAC as well as long-term survival; they reported that a decrease in the levels of glycine after NAC was associated with long-term survival.^10^ In the present case report, the glycine peak was identified on pre-treatment MRI but not on post-treatment MRI after the first cycle of NAC; however, it is not clear whether this was related to early change in tumor metabolism after treatment or to a technical issue.

Glycine is an inhibitory neurotransmitter and co-agonist at glutamatergic N-methyl-D-aspartate receptors; it has been suggested to be an *in vivo* biomarker for brain tumor malignancy.^1,11–14^ Glycine has been found at higher concentrations in high-grade brain tumors relative to low-grade tumors, both *ex vivo* and *in vivo*.^15^ However, the specific role of glycine in malignancy and in different cancers is currently unknown. Glycine is a small, non-essential amino acid, which can be derived from glycolysis through its precursor serine from 3-phosphoglycerate, or from choline by oxidation of choline to betaine which is further converted to glycine.^16^ Thus, increased levels of glycine in cancer patients may result from altered glycolysis and/or metabolism of choline.

*In vivo* MRS requires a high magnetic field strength to quantify small metabolites. While most clinical MRS measurements are performed using 1.5 T devices, the advent of 3.0 T MR systems has improved MRS signal-to-noise ratio, allowing a higher spectra resolution. The glycine peak has a spectral overlap with *myo*-inositol in a 1.5 T magnetic field; however, the glycine peak is more easily distinguished at 3.0 T.^13,14^ Ultra-high fields MR systems (>3.0 T) can provide even better spectra but to date they are used only for scientific research and their clinical usefulness is still under debate because of their very high costs, several technical challenges, and concerns regarding their biological effects.^17^

In conclusion, we report a *HER2*-overexpressed breast cancer patient who showed elevated glycine concentrations on pre-treatment *in vivo* MRS. Since high glycine levels have been associated with poor prognosis in breast cancer patients on *ex vivo* MRS, further studies are needed to understand the role of glycine in cancer progression.

## Learning points

- Breast MRS is a promising non-invasive diagnostic method that can detect and quantify endogenous tissue metabolites.
- High glycine concentrations have been described in high-grade brain tumors; however, the role of glycine in malignancy and in different cancers is currently unknown.
- Studies using *ex vivo* MRS have described high glycine concentrations in breast cancer and have suggested this to be a marker of poor prognosis.
- *In vivo* MRS performed with higher magnetic field strength devices (>1.5 T) will allow better distinction of glycine concentrations and enable improved assessment of prognosis and treatment response in breast carcinomas.
